# Etanercept may induce neurosarcoidosis in a patient treated for rheumatoid arthritis

**DOI:** 10.1186/1471-2377-13-212

**Published:** 2013-12-28

**Authors:** Cécile-Audrey Durel, Elodie Feurer, Jean-Baptiste Pialat, Emilie Berthoux, Roland D Chapurlat, Cyrille B Confavreux

**Affiliations:** 1 INSERM UMR 1033 – Université de Lyon, Department of Rheumatology, Hôpital Edouard Herriot, 5, Place d’Arsonval, Hospices Civils de Lyon, Lyon 69003, France; 2 Department of Internal Medicine, Hôpital Edouard Herriot, Université de Lyon, Hospices Civils de Lyon, Lyon 69003, France; 3 INSERM UMR 1033 – Université de Lyon, Department of Radiology, Hôpital Edouard Herriot, Hospices Civils de Lyon, Lyon 69003, France

**Keywords:** Neurosarcoidosis, Rheumatoid arthritis, Etanercept, Facial palsy, TNF alpha blockers

## Abstract

**Background:**

TNFα blockers have drastically improved rheumatoid arthritis prognosis by preventing joint destruction in DMARD resistant patients. Altering cytokine balance in immune diseases may expose to paradoxical adverse events.

**Case presentation:**

We present the case of a 40-year-old woman, with a confirmed erosive and seropositive RA, successfully treated by TNFα blocker (etanercept) for seven years, and who developed a severe neurosarcoidosis. She had lymphocytic meningitis, bilateral peripheral facial paralysis and anosmia, associated with bilateral hilar lymph nodes, papilloedema, anterior uveitis and elevated serum angiotensin-converting enzyme level. Magnetic resonance imaging showed a bilateral thickening of the Gasser’s ganglia walls and enhanced signal of the vestibulocochlear, the facial and the proximal portion of trijeminal nerves.

**Conclusion:**

This case raised the issue of the imputability of etanercept in the development of neurosarcoidosis. Neurological symptoms onset in patients on TNFα blockers should lead to exclude infections, induced lupus but also paradoxical neurosarcoidosis.

## Background

Tumor necrosis factor alpha (TNFα) blockers have drastically improved the prognosis of rheumatoid arthritis by preventing joint destruction in DMARD resistant patients. We present the case of a RA patient on etanercept who suddenly developed neurosarcoidosis.

## Case presentation

**Figure 1:**
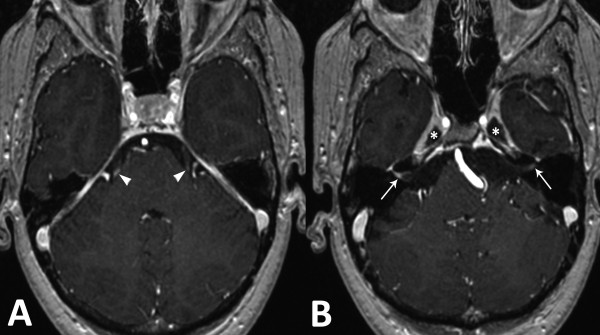
**Axial MRI images of the brain.** Post gadolinium enhanced T1 weighted sequence on initial exam shows **(A)** bilateral focal enhancement of the proximal portion of both trijeminal nerves (arrowheads), **(B)** bilateral enhancement of the vestibulocochlear and facial nerves, more pronounced on the right side (arrow) and bilateral thickening of the Gasser’s ganglia walls (*).

A 40-year-old woman suffered from severe erosive rheumatoid arthritis with positive rheumatoid factor (251 UI/ml [normal <25 UI/ml]) and positive anti-cyclic citrullinated antibodies (64UA/ml [normal <2.9 UA/mL]). In 2004, etanercept was added to methotrexate to control her disease. In 2008, because of hepatic cytolysis, methotrexate was stopped but etanercept monotherapy maintained the remission. In 2012, she suddenly presented an isolated left peripheral facial palsy. Magnetic resonance imaging (MRI) showed a contrast-enhancement of the facial nerve. Rapidly, her clinical status worsened with bilateral facial palsy, anosmia, weight loss, dyspnea, eyesight deficiency, eye and mouth dryness. Ophthalmological examination revealed bilateral Bell’s phenomenon and left papilloedema with anterior uveitis. Biological analysis revealed elevated serum calcium level (2.56 mmol/L), hyperproteinemia (90 g/L), cytolysis (1.5 N) and cholestasis (4N). Cerebral MRI showed an enhanced signal of cranial nerves on the post enhanced T1-weighted sequences (Figure 1A, B). C-reactive protein was normal. Serologies for Human Immunodeficiency Virus (HIV), hepatitis B, C, syphilis and Lyme were negative. Cerebral fluid analysis showed a lymphocytic meningitis (hyperproteinorachia 0,62g/L and normoglycorrhachia) without oligoclonal band. Polymerase chain reactions of human herpes virus and enterovirus were negative. Chest CT-scan found bilateral hilar and mediastinal lymph nodes. The bronchoalveolar lavage brought a lymphocytic alveolitis (53%) with a high CD4/CD8 lymphocytic ratio of 4.65. Quantiferon® assay and mycobacterium culture remained negative. The 18F-fluoride PET-CT supported the diagnosis of sarcoidosis showing a fixation of hilar lymph nodes, parotid and submandibulary glands without hepatic or other abnormal fixations. Normal serum complement and absence of anti-nuclear antibodies ruled out TNFα induced neurological lupus. Etanercept was stopped and intravenous steroid boluses were initiated (15 mg/kg per day for 3 days) followed by oral prednisolone (1 mg/kg per day). For technical reason, transbronchial lung biopsy was delayed after 1 month of steroid and remained not contributive. Nevertheless, diagnosis of neurosarcoidosis was further supported by an increased level of angiotensin-converting enzyme in serum (77 UI/L [normal value < 68 UI/L]). After 3 months, thanks to a normalization of the liver function, methotrexate (25 mg/week) was added to treat both RA and neurosarcoidosis without another secondary hepatic cytolisys. One year later, she remained on methotrexate and prednisone 20 mg/day: eyesight has completely recovered but not the facial paralysis. Brain MRI showed normalization.

## Conclusions

Paradoxical neurosarcoidosis appeared as the RA was in complete remission on etanercept monotherapy for several years. Since we did not obtain histological confirmation, this case is not a definite neurosarcoidosis according to Zajicek’s criteria [1]. The delay of one month to perform biopsy while already on steroids may be the reason for non contributive histology. Nevertheless, we eliminated other neurological diseases and the diagnosis was supported by neurological impairment (lymphocytic meningitis, bilateral peripheral facial paralysis, anosmia, eyesight deficiency, papilloedema) associated with anterior uveitis, eye and mouth dryness, weight loss, hypercalcemia and hyperproteinemia. The presence of evidences for systemic sarcoidosis criteria with bilateral hilar lymph nodes, lymphocytic alveolitis, and elevated serum angiotensin-converting enzyme level classified this case as probable [1]. More recently, chest radiograph and angiotensin-converting enzyme of Zajicek’s criteria have been shown to be of insufficient diagnostic value by Marangoni and replaced by chest high resolution computed tomography and broncheoalveolar lavage, with a CD4/CD8 lymphocytic ratio higher than 3.5, of better positive predictive value [2]. According to these revised criteria, our case is still considered as probable [2].

TNFα blockers have already been associated in some cases with “paradoxical” systemic sarcoidosis [3] but only two cases of neurosarcoidosis have been reported and none with etanercept. One occurred in another RA female patient treated by infliximab and methotrexate [4]. The second one refers to a man on adalimumab for an ankylosing spondylitis, who developed seizures [5]. These three cases raise the issue of TNFα blockers imputability on the onset of neurosarcoidosis. Interestingly, some TNFα-blocker-induced sarcoidosis patients did not relapse after rechallenging [6]. However, there are biological arguments to support imputability of TNFα blockers to sarcoidosis onset as a “class effect” rather than a drug specific phenomen regarding sarcoid-like granulomatous disease occurring during all three anti-TNF therapies. About 40 cases of sarcoid-like granuloma development during anti-TNF therapy have been actually reported in the literature. First, the key pathophysiological feature of sarcoidosis is granuloma. It aims at isolating pathogens and restricting inflammation. As sarcoidosis preferentially involves skin, lungs and eyes, one mechanism relies on the direct exposure to environmental antigens [7]. Thus the induced immunosuppressive condition may favor development of microorganisms involved in sarcoidosis development (*Propionibacterium acnes* or *granulosum)*. A second mechanism in sarcoidosis is that granulomas are characterized by local proinflammatory activated CD4 + T lymphocytes with Th1 profile (IFNγ, IL2) stimulating TNFα production by macrophages. Sarcoidotic patients have simultaneously systemic anergy due to the suppressive abilities of regulatory CD4 + CD25 + FoxP3 T lymphocytes partly triggered by TNFα. Thus TNFα blockade, independently to its anti-inflammatory effect, may be able to stimulate Th17 pathway (IL17, IL23) [8] and to alter T-reg lymphocyte subpopulations [9].

By contrast some studies reported refractory sarcoidosis cases successfully treated by TNFα blockers especially adalimumab and infliximab [10]. Indeed, monoclonal TNFα blockers antibodies are considered to be more effective to block granuloma formation rather than etanercept which partially preserves the mechanisms leading to granuloma formation. This could explain a lack of efficacy of etanercept in granulomatous diseases (e.g. refractory sarcoidosis, crohn’s disease).

In addition to paradoxical neurosarcoidosis, other neurological adverse events have been reported with the use of TNFα blockers such as multiple sclerosis [11], polycranial neuritis, chronic inflammatory demyelinating polyradiculoneuropathy and multifocal motor neuropathy [12]. TNFα blockers may trigger the demyelinating process which can evolve independently afterwards [13]. Thus physician should pay a particular attention to patients on TNFα blocker therapy who present any new neurological symptoms.

In conclusion, this case of paradoxical neurosarcoidosis induced by TNFα blockade in a RA patient underlines the risk to destabilize autoimmune profile when using targeted therapies and promote unexpected immune disease.

## Consent

Written informed consent was obtained from the patient for publication of this Case report and any accompanying images. A copy of the written consent is available for review by the Editor of this journal.

## Competing interests

The authors declare that they have no competing of interests.

## Authors’ contributions

EF, CAD, JBP, EB, RDC and CBC diagnosed and treated the patient, contributing equally to writing and revising the manuscript. All authors read and approved the final manuscript.

## Pre-publication history

The pre-publication history for this paper can be accessed here:

http://www.biomedcentral.com/1471-2377/13/212/prepub
